# iSupport for rare dementias: a mixed-methods non-randomised feasibility study of an online self-help programme for carers

**DOI:** 10.1186/s40814-025-01639-z

**Published:** 2025-04-30

**Authors:** Bethan Naunton Morgan, Gill Windle, Carolien Lamers, in collaboration with the Rare Dementia Support (RDS) Impact project research team

**Affiliations:** 1 School of Psychology and Sports Science, Brigantia Building, Bangor University, Penrallt Road, Bangor, UK; 2 School of Health Sciences, Bangor University, Bangor, UK; 3 North Wales Clinical Psychology Programme, Bangor University, Bangor, UK

**Keywords:** Rare dementia, eHealth, Internet, Caregiver, Carer, Supportive intervention, Adaptation, iSupport

## Abstract

**Background:**

iSupport for dementia carers is an online education and self-care programme developed by the World Health Organisation for carers of people with the most common forms of dementia (Alzheimer’s disease and vascular dementia). iSupport for rare dementias (RDC) is the first adaptation designed specifically to address the challenges faced by carers of individuals with rare dementias (frontotemporal dementia, posterior cortical atrophy, primary progressive aphasia or Lewy body dementia).

**Methods:**

A 12-week mixed-methods non-randomised feasibility study assessed the feasibility of recruitment and participant retention, the feasibility of outcome measures and the acceptability of iSupport RDC. Participants were recruited through the Rare Dementia Support Network (target *N* = 30). Data were collected through online interviews and self-report, including pre and post-intervention measures of depression, anxiety, burden and resilience. A modified version of the NoMAD questionnaire evaluated acceptability of implementation. Scores range from 0 to 4 with > / = 2.5 indicating acceptability. Usability was assessed through self-report and data from Blackboard.

**Results:**

Thirty-four (13 males and 21 females) carers of people with frontotemporal dementia, posterior cortical atrophy, primary progressive aphasia or Lewy body dementia consented to the study and given access to iSupport RDC, hosted online by Blackboard Learn. Their ethnicity was reported as white and their mean age was 64.2 (range 35–86). *N* = 24 completed pre and post outcome measures, *N* = 10 completed pre-intervention and then withdrew, *n* = 4 reporting technical difficulties (70.6% completion rate). There were no missing responses. *N* = 20 completed 3 of the 5 iSupport RDC modules; *n* = 13 completed five. *N* = 4 could not access due to technical difficulties. Technical difficulties meant the data from Blackboard Learn were not obtained. The NoMAD total score (3.5) indicated iSupport RDC was acceptable. Qualitative analysis from *n* = 19 participants revealed themes of ‘technical difficulties’ (*n* = 10), ‘useful and informative’ (*n* = 7), and ‘provide at point of diagnosis’ (*n* = 5).

**Conclusions:**

Recruitment targets were met but there were limitations in sample diversity. The extent of attrition warrants strategies to ensure retention to future studies, including testing online interventions on different internet browsers and operating systems. The favourable response to iSupport RDC from the participants indicates its potential as a valuable resource for supporting carers dealing with rare dementias.

**Supplementary Information:**

The online version contains supplementary material available at 10.1186/s40814-025-01639-z.

## Key messages regarding feasibility

- What uncertainties existed regarding the feasibility?
  The functionality of iSupport for Rare Dementias (RDC) was unclear. Questions were raised about the potential challenges in participant recruitment, the reception of the intervention, and the overall acceptability of the programme among carers of people with rare dementias.
- What are the key feasibility findings?
  Noteworthy insights were derived from the feasibility study, indicating a discernible level of interest and active participation among the targeted audience. Successful recruitment was achieved, and the intervention received positive feedback from carers. However, IT related difficulties were reported by 10 participants and resulted in four participants withdrawing, therefore more thorough testing of online interventions is recommended on different internet browsers and operating systems.
- What are the implications of the feasibility findings for the design of the main study?
  The favourable response to iSupport RDC from the participants underscores its potential as a valuable resource for supporting carers dealing with rare dementias. In planning a larger feasibility study, a critical focus should be placed on strategies to maintain participant engagement over an extended period. The incorporation of supplementary support mechanisms, such as regular check-ins with the researcher to offer technical support could be beneficial to address the identified concerns and contribute to the overall efficacy of the intervention. Additionally, the intervention should be thoroughly tested prior to recruitment for any anomalies on different computer systems.

## Background

The burden of dementia care is a well-established global health concern, affecting millions of people and their families [1]. While considerable attention has been devoted to the care of people with common forms of dementia, such as Alzheimer’s disease, there is a growing awareness of the unique challenges faced by those caring for individuals with rare dementias [2]. Rare dementias include over one-hundred neurodegenerative disorders, including frontotemporal dementia (FTD), posterior cortical atrophy (PCA), primary progressive aphasia (PPA), and Lewy body dementia (LBD) and each characterised by different symptoms and clinical presentations [3–5]. These rare dementias are more likely to occur in people under the age of 65 and have symptoms that differ from the memory impairments associated with Alzheimer’s disease [5]. The symptoms can include changes to behaviour, vision, and movement [2, 5, 6]. For the purposes of this study, the rare dementias referred to are FTD, PCA, PPA and LBD.

The experiences for carers of people living with rare dementias can be more difficult due to a lack of understanding by services regarding these conditions and limited specialised support [4], which may cause an additional hurdle for their carers [2, 4, 7]. As the prevalence of rare dementias becomes increasingly recognised, the need for tailored and evidence-based support interventions for these carers has grown [4, 8].

Accessing face-to-face support can be challenging for carers of people with rare dementias due to limited availability of specialised services, and due the low prevalence of the conditions, the formation of local support groups is not always viable [4]. The resulting isolation can intensify carer stress levels and limit opportunities for information exchange and emotional support [2, 8]. However, online supportive interventions have emerged as a practical solution to bridge this gap. These digital platforms remove the obstacle of geographical barriers, offering easily accessible resources, fostering connections with other carers facing similar challenges, and delivering tailored information and guidance specific to rare dementia types [9–11].

‘iSupport’ was developed by the World Health Organization (WHO) [12]. It is an online resource for carers of people with dementia designed to improve their knowledge about dementia, to enhance their coping skills and the quality of life for both the carer and the person with dementia [12, 13]. However, iSupport was designed for the more typical/common conditions of Alzheimer’s Disease and Vascular Dementia, therefore not addressing the unique challenges of carers dealing with rarer forms of the disease.

Recognising the limitations of iSupport for rare dementia types such as frontotemporal dementia, posterior cortical atrophy, primary progressive aphasia, or Lewy body dementia, a specialised adaptation named ‘iSupport for rare dementia carers’ (iSupport RDC) was created [14]. This adaptation used co-design methods involving carers of people with rarer dementias and healthcare professionals as recommended in the WHO adaptation guidelines [15]. iSupport RDC addresses the unique challenges and requirements faced by carers of individuals with rare dementias, and includes information of the different symptoms, the challenges related to a potential younger age of onset and offers tailored support and guidance specific to rare dementias [14]. The purpose of this study was to examine the feasibility of iSupport RDC ahead of further research into its effectiveness and implementation.

## Methods

The aims were to undertake a 12-week mixed-methods feasibility study of iSupport RDC and examine: (1) eligibility, recruitment, retention and adherence; (2) the suitability of outcome measures; (3) the acceptability and use of iSupport RDC. A non-randomised approach was chosen as treatment effects were not evaluated and this approach facilitated an in-depth exploration of iSupport RDC. This paper follows guidance from the CONSORT extension to pilot and feasibility trials [16] (Additional file 1).

### Ethics

Ethical approval was granted from University College London (UCL) as a part of the larger study titled the ‘Rare Dementia Support Impact project’ (The impact of multicomponent support groups for those living with rare dementias, (ES/S010467/1)), (UCL ethics number: 8545/004). Ethical approval was also obtained from Bangor University School of Psychology Ethics and Research Committee (Ref. number: 2020–17057).

### iSupport RDC

iSupport RDC is an online support package and consists of five modules: (1) what is dementia, (2) being a caregiver, (3) caring for me, (4) providing everyday care and (5) dealing with symptoms and behaviour changes. Each session includes interactive activities that give carers information and feedback. iSupport RDC is designed to be flexible and accessed from the carer’s homes at a time that suits them.

To test its feasibility and acceptability, iSupport RDC was hosted on Blackboard Learn, an online teaching, learning and collaboration application. Blackboard Learn was selected to host iSupport RDC following consultation with the University IT department. This platform was chosen for its robust security features, user-friendly interface, and widespread institutional support, ensuring data protection and accessibility for participants. Additionally, Blackboard Learn’s ability to track user engagement aligned with the study’s objectives to evaluate the feasibility and usability of the iSupport RDC platform. Its established use within the university infrastructure further facilitated technical support and streamlined the setup process. As Blackboard Learn normally does not allow anonymity due to its collaborative nature (e.g. users can see the email identifiers of each other), adjustments were made to ensure participant privacy in this research, which consisted of the creation of separate iSupport modules for each participant. Participants were provided with individual usernames and randomised passwords by the lead author to access iSupport RDC.

### Participant characteristics

The study involved people who were currently providing care for someone with a rare form of dementia. To ensure the appropriateness of the participants, the study used the following inclusion and exclusion criteria:

Inclusion criteria:

1. Ability to provide informed consent for participation in the research.
2. Age 18 years or older.
3. Currently caring for someone diagnosed with frontotemporal dementia (FTD), posterior cortical atrophy (PCA), primary progressive aphasia (PPA) or Lewy body dementia (LBD). These specific conditions were chosen due to their unique characteristics, as they do not initially present with the memory-related symptoms typically associated with more common forms of dementia.

Exclusion criteria:

1. Inability to comprehend written English, as this is essential for participants to effectively engage with iSupport RDC and communicate their opinions of it.
2. Lack of access to a computer or tablet with an internet connection.

While the inclusion and exclusion criteria ensured participants could engage with the iSupport RDC platform, they may have introduced biases by excluding non-English speakers and those without digital access. However, the sample represents caregivers likely to benefit from digital interventions, reflecting a relevant subset of the broader population of people caring for someone with a rare dementia.

### Sampling procedures

Participants were recruited through the Rare Dementia Support (RDS) network as a part of the larger RDS Impact study [7]. RDS is a service for people living with rarer dementias provided by University College London [3]. As part of the sign-up process to become a member of the RDS network, people can agree to be contacted about participating in research.

Recruitment procedures followed the protocol for the RDS Impact study [7]. Potential participants were contacted by email and offered the opportunity to take part in the study. Once they expressed an interest in participating, they were sent the participant information sheet and consent form. A video-call was arranged where an audio-visual recording of the consenting process took place, whereby the researcher read the consent form to the participant and video-recorded their stated verbal consent to each point on the form. After the consent meeting, participants were sent login details and instructions on how to log in to iSupport (Additional file 2). Verbal consent was prioritised over written consent in this study to align with the consent procedures established by the larger RDS Impact Study [7], which recruits both people living with dementia and their carers. Given the inclusion of participants living with dementia in the RDS Impact Study, verbal consent was used to assess participants’ capacity to consent in real-time, ensuring they understood the study and could provide informed consent. This approach is consistent with ethical guidelines that prioritise participant’ autonomy while accommodating potential cognitive impairments.

### Sample size

While a sample size calculation is not required for a feasibility study, it is important to justify the size [17]. Recommendations for sample sizes of feasibility trials vary from 10 to 75 participants per group with a median of 30 to 36 [18]. This feasibility study aimed to recruit 30 participants, as suggested by Lancaster et al. [19] and is the median number found by Lewis et al. [18]. A small sample size for feasibility studies in the early stages of testing interventions, means that the results are available sooner so that further adaptations can be made to the intervention [17, 20]. Starting the testing of an intervention with a smaller sample size enables an understanding of the elements of the intervention that do not work well while requiring fewer resources to run [20].

### Measures

#### Primary outcome measures

The primary outcomes were feasibility and acceptability of the adapted iSupport intervention. Feasibility was assessed using recruitment data and other measurement tools.

1. Feasibility of recruitment: assessed by the number of participants willing to take part in the study. Demographic information was collected (e.g. age, gender, relationship to the person with dementia) to determine the variance of participants’ characteristics.
2. Feasibility of the measurement tools: The feasibility of the measurement tools (used to assess the secondary outcomes) was investigated using the responses to the questionnaires. Missing questions, late responses and time taken to complete the questionnaires were used to determine the suitability of the measures.
3. Acceptability of the intervention: To investigate the acceptability of the iSupport adaptation, data on the number of times iSupport had been accessed and the number of modules completed was to be recorded. Participants’ feedback was also invited on the acceptability of the intervention at the end of the programme. This was collected using a modified version of the NoMAD questionnaire [21] based on the Normalisation Process Theory (NPT) [22] and was available in English and Welsh (Additional file 3).

NPT has been created for use in the development and evaluation of complex interventions [22]. It considers the components necessary for the implementation and integration of an intervention into everyday life. The NoMAD questionnaire measures four constructs of NPT [21–23]:

1. Coherence: This refers to understanding the intervention’s purpose and how it fits into existing practices.
2. Cognitive Participation: this involves active engagement and commitment to implementing or using the intervention.
3. Collective Action: This is the practical efforts made to enable the intervention to become a part of routine practice.
4. Reflexive Monitoring: Ongoing assessment of the intervention’s benefits and costs by those involved.

The NoMAD questionnaire looks at the normalisation of interventions from the perspective of professionals who will be implementing the intervention as well as service users [21, 23]. For the purpose of this study, the ten questions aimed at professionals were removed, leaving thirteen questions from the NoMAD questionnaire relevant to the carers. Answer options were on a Likert scale (e.g. “I understand the purpose of iSupport for Rare Dementias: 0-Strongly agree- 4-Strongly disagree”). A score above 2.5 is seen as a positive result. To address other aspects of intervention implementation, the NoMAD questionnaire can be adapted by adding or rephrasing questions to make it relevant for different stages of testing or different areas of interest [21, 23]. Since the questionnaire was designed with the flexibility to remove irrelevant questions, the guidelines on interpreting the scores advise to use frequencies or means to summarise the data [21, 23]. Four additional questions were added on the usability of iSupport RDC: “Did you have problems accessing the iSupport website?—If yes please describe-”, “How much of iSupport did you complete?”, “Did you access the iSupport website or view the PDF version?”, and “Do you have any additional feedback on iSupport for Rare Dementias?” (Additional file 3).

#### Secondary outcome measures

An umbrella review [10], found that online interventions for carers of people with dementia are linked to reductions in carer depression, anxiety, burden, and increases in self-efficacy scores. The mental health outcomes were selected for this feasibility study since the review found them to be among the most commonly used in research with carers of people with dementia. Resilience was selected instead of self-efficacy, as resilience is a broader concept that encompasses self-efficacy and other positive attributes such as self-esteem [24, 25]. While the Zarit Burden Interview [26] was specifically developed for carers, the other measures were not. The measures were available in English and Welsh in line with Bangor University’s bilingual policy.

1. Depression was assessed with the Center for Epidemiologic Studies Depression Scale (CES-D-10) [27]. This is a 10-item self-report measure of depression that has demonstrated high internal consistency, reliability, and validity [28]. The responses on this measure range from 0 (rarely or none of the time) to 3 (most or all of the time) and range from 0 to 30, with higher scores indicating more depressed feelings.
2. Anxiety was assessed using the Generalized Anxiety Disorder questionnaire (GAD-7) [29]. This is a commonly used measure of anxiety symptoms experienced over the previous two weeks. There are seven items, the responses are on a Likert scale ranging from 0 (not at all) to 3 (nearly every day) and the overall scores range from 0 to 21. GAD-7 shows high reliability, validity, and internal consistency [29]. Scores ranging from 0 to 4 signify minimal anxiety, scores between 5 and 9 represent mild anxiety, scores spanning from 10 to 14 denote moderate anxiety, and finally, scores falling within the range of 15 to 21 indicate severe anxiety.
3. Carer burden was assessed using the Zarit Burden Interview (ZBI) [26]. This is a shortened version of with 12 items (instead of 22), which has shown to be a faster and valid and reliable measure of burden in carers of people with dementia [30]. Participants respond to 12 statements using a Likert scale ranging from 0 (never) to 4 (almost always). Overall scores range from 0 to 48 and higher scores indicate higher feelings of burden. No to mild burden is demonstrated with a score of 0–10, mild to moderate burden is 10–20, and high burden is a score of 20 or above.
4. Resilience was measured using the Resilience Scale-14 (RS-14) [31], which is a 14-item version of the original 25-item resilience scale. The response options range from 1 (Strongly disagree) to 7 (strongly agree) with overall scores ranging from 14 to 98 and this measure shows high construct validity and internal consistency [31]. People scoring between 14 and 30 are classified as having a very low level of resilience, scores falling within the range of 31 to 48 indicate a low level of resilience, scores spanning from 49 to 63 denote an average level of resilience, scores ranging from 64 to 81 signify a high level of resilience, and people scoring between 82 and 98 are identified as having a very high level of resilience [31].

### Procedure

Demographic data were collected pre-intervention, before participants accessed iSupport. Data for the secondary outcomes were collected online, pre- and post (after three months of using iSupport, counting from when they were sent the login details for iSupport) intervention. Participants’ evaluation data were gathered after participants had three months access to iSupport. The system usability data were to be collected by the website host (Blackboard Learn) throughout the intervention period.

### Data analysis

#### Quantitative data

For the quantitative data, scores before and after using iSupport were compared. Data were analysed descriptively without statistical tests due to the nature of feasibility studies requiring small sample sizes that do not have the power for these tests [32]. However, as the data were not normally distributed, the median scores were used, and confidence intervals calculated. Percentage changes were calculated for each measure, pre- and post-intervention, thus making the changes between measures comparable.

#### Qualitative data

The qualitative questionnaire data were analysed using content analysis following the guidelines from Bengtsson [33]. The process involves categorising feedback based on emerging themes, known as coding and categorisation. By analysing these codes, researchers aim to identify recurring patterns or themes within the content. Then the findings are interpreted in relation to the research question, allowing for the drawing of meaningful conclusions. Throughout the process, Bengtsson [33] highlights the significance of ensuring the rigor of the analysis, suggesting techniques such as inter-coder reliability checks to maintain accuracy and credibility. Therefore, the initial coding and categorisation step was completed by the lead author (BNM) and validated by co-authors (GW and CL). The outcomes from this feasibility study were used to discuss the potential for the adapted version of iSupport to undergo further testing and whether the current methods could be effective on a larger scale.

#### Researcher characteristics and reflexivity

When completing qualitative data analysis, it is important that researchers are aware of potential biases that may affect their interpretation of the data [34]. All three researchers are white females with backgrounds in psychology and educated to university level. Their interpretations might be influenced by their cultural lenses, impacting how they perceive certain behaviours, expressions, or experiences depicted in the data. The lead author (BNM) has experience in dementia research and as a carer in a dementia nursing home. The second author (GW) has experience in dementia research. The third author (CL) has experience in dementia research and working with people who have dementia and their carers, as a clinical psychologist. These experiences could potentially influence their interpretations based on real-life encounters and emotions experienced in these different settings.

Each researcher may unintentionally have interpreted the data based on their preconceived notions, beliefs, or past experiences. This could influence how they analysed and understood the narratives or information gathered during the study. The research team is not diverse in terms of gender, ethnicity or level of education, which may have biased the interpretation of the data. However, researcher openness and reflections during data analysis meetings aimed to address these challenges and provide a better understanding of the research findings [34].

### Progression criteria

While many feasibility studies use predefined progression criteria to decide whether to move to larger trials, some researchers suggest a more flexible, thorough approach since there are no clear guidelines on how to set these criteria [35, 36]. Consequently,* s*pecific progression criteria were not established because the aim was to comprehensively assess various aspects of the study without limiting the scope. Considering all findings related to the feasibility of recruitment, the feasibility of the measures, and the acceptability of the intervention, ensured a thorough evaluation. This approach allowed the gathering of nuanced insights and addressed any potential issues holistically, providing a solid foundation for future research and ensuring that the conclusions were robust and well-informed.

## Results

### Feasibility of recruitment

**Fig. 1:**
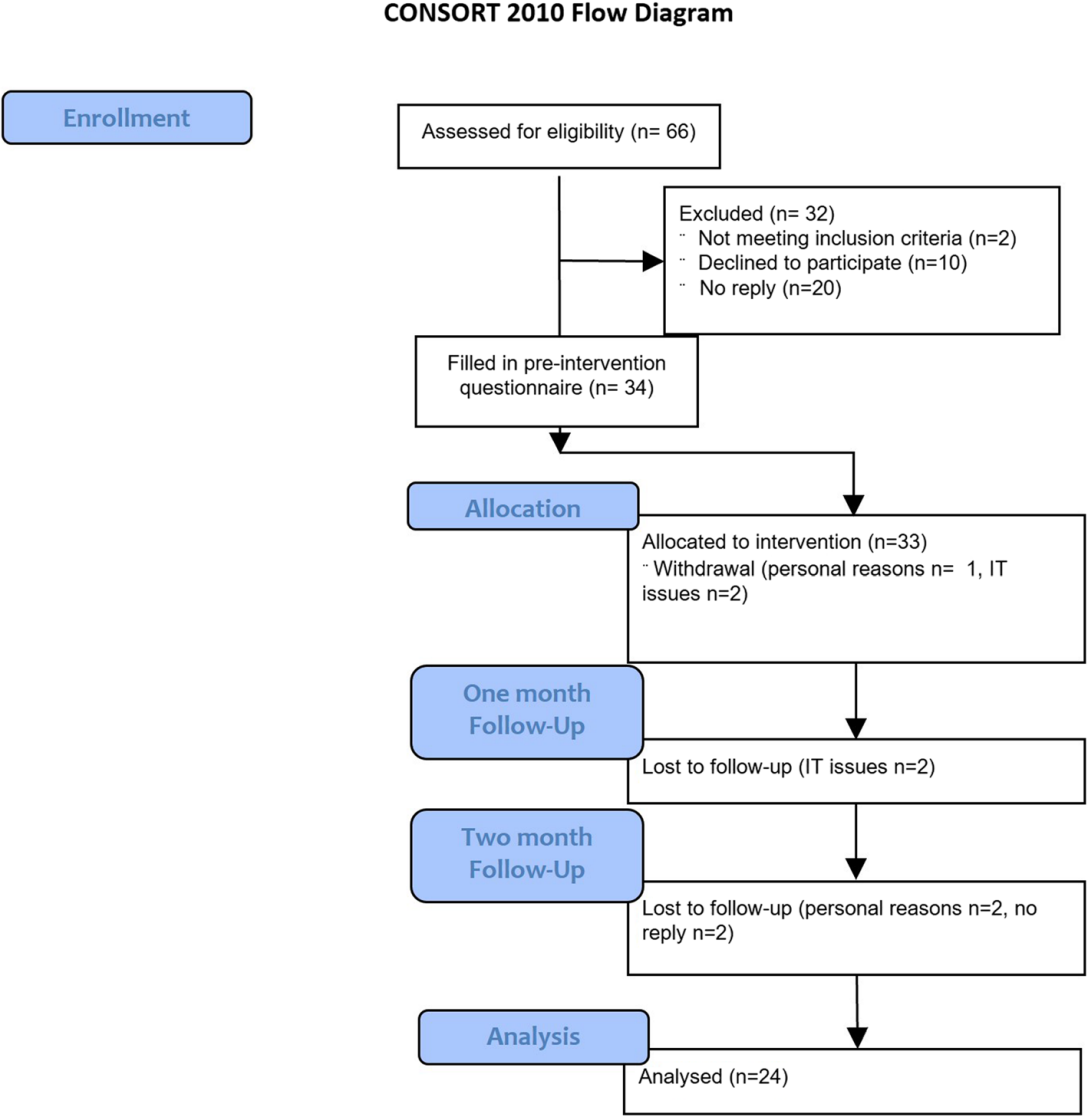
CONSORT flow chart of procedures and number of participants at each stage

Recruitment took place over four weeks between 09.05.2023 and 06.06.2023. Figure 1 shows that sixty-six potential participants were invited to take part, 36 expressed an interest, one expressed interest but did not reply on follow up, one did not meet the inclusion criteria, 20 did not reply and 10 declined the invitation. A total of 34 participants consented to take part in the study, which was half of those invited. Thirteen were male and twenty-one female and the average age was 64.2 (35–86). There was an absence of ethnic diversity in the participants (Table 1).

**Table 1:** Demographic data for participants

Variable	Frequency (%)	
	Pre-Intervention	Post-intervention
Gender		
Male	13 (38.2)	11 (45.8)
Female	21 (61.8)	13 (54.2)
Age		
30–49	4 (11.8)	3 (12.5)
50–59	6 (17.6)	5 (20.8)
60–69	15 (44.1)	11 (45.8)
70–79	7 (20.6)	3 (12.5)
80–89	2 (5.9)	2 (8.3)
Ethnic group		
White British	30 (88.2)	20 (83.3)
Irish	2 (5.9)	2 (8.3)
Other (Australian and White European)	2 (5.9)	2 (8.3)
Relationship to PwD		
Spouse	28 (82.4)	20 (83.3)
Child/parent	5 (14.7)	4 (16.7)
Sibling	1 (2.9)	0 (0.0)
Education		
Postgraduate qualification	6 (17.6)	4 (16.7)
Degree level	14 (41.2)	9 (37.5)
A-level, Baccalaureate	4 (11.8)	4 (16.6)
Post-secondary certificate or diploma	3 (8.8)	2 (8.3)
GCSE’s/O levels	2 (5.9)	1 (4.2)
Other	5 (14.7)	4 (16.7)
Type of dementia		
Posterior cortical atrophy	11 (32.4)	7 (29.2)
Primary progressive aphasia	14 (41.2)	10 (41.7)
Frontotemporal dementia	6 (17.7)	6 (25.0)
Lewy body dementia	3 (8.8)	1 (4.2)

Ten participants did not complete the study. Four reported IT related difficulties and decided to withdraw, two gave personal reasons for their withdrawal and four gave no reason (Fig. 1). This gave a 70.6% completion rate.

The 10 participants who did not complete the post-intervention measures had higher pre-intervention median scores for anxiety, depression, and burden, but scored higher on resilience than the participants who completed the study (Table 2). Eight were female (80%) with an average age of 64 and 50% had a degree level qualification or higher.

**Table 2:** Differences in median pre-intervention measures (anxiety, depression, burden, and resilience) of participants who withdrew and those who completed the study with interquartile ranges (IQR)

	GAD Pre Mdn (IQR)	CES Pre Mdn (IQR)	ZBI Pre Mdn (IQR)	RS Pre Mdn (IQR)
Withdrawn	9 (6)	14 (9)	26 (7)	82 (26)
Completed	6 (6.8)	12 (7.5)	22 (8.5)	75 (19)

### Feasibility of measurement tools

Twenty-four participants completed all measures, pre- and post-intervention. Ten people completed the pre-intervention measures only and one person completed all pre- intervention measures but only the GAD and CES-D measures at post-intervention, due to a recent bereavement. None of individual questions of any of the four measures had missing responses.

The average time for the 34 participants to complete the pre-intervention questionnaires (demographics, ZBI, CES-D, GAD, RS) was 1 h and 19 min, ranging from 3 min to 24 h and 50 min. However, these results were skewed by two participants who were not able to complete the questionnaire in one sitting demonstrating the unpredictable nature of caring for someone with a rare dementia, where participants’ availability and caregiving demands fluctuate. Consequently, median scores were calculated instead, the median time for the pre-intervention questionnaires was 7.6 min (IQR: 562) and for the 23 participants who completed the questionnaires post-intervention (ZBI, CES-D, GAD, RS and NoMAD) the median time was 8 min (IQR: 201).

### Acceptability of the intervention

The usability data provided by the Blackboard Learn platform were unreliable and gave conflicting results, for example, the system reported that one participant had not logged in at all, but also spent time on the introduction module of iSupport RDC. This discrepancy suggests potential issues with data recording or syncing on the platform. In future studies, it would be beneficial to implement additional monitoring tools to cross-check platform data, such as manual tracking or more frequent system updates. Additionally, using platforms with more robust tracking capabilities, or conducting real-time data verification, could help improve the accuracy of usability data and reduce inconsistencies.

Consequently, participant engagement with iSupport could only be assessed using self-report data. Carers’ reports indicated that 23 (68%) of the 34 participants with access to the Blackboard Learn used iSupport during the study period. Four participants, who had difficulty accessing Blackboard Learn, were provided with a PDF version of iSupport. As a part of the post-intervention questionnaires, participants were asked how many modules they completed. Twenty participants (59%) reported they completed at least three modules of the intervention and 13 (38%) participants said they completed all five modules. The four participants who had access to the PDF version reported to have completed all five modules of iSupport.

Participants scored iSupport RDC an average 3.5 out 5 on the NoMAD questionnaire (Additional file 3). Mean scores for each of the four components of NPT were calculated (Table 3).

**Table 3:** The four components of NPT, their mean (M) scores and standard deviations (SD)

Coherence M (SD)	Cognitive participation M (SD)	Collective action M (SD)	Reflexive monitoring M (SD)
4.1 (0.8)	3.1 (1.5)	3.2 (0.9)	3.5 (1.2)

Coherence (NPT Component 1) was assessed through questions 4–6. This was the highest scoring NPT component; participants gave it an average score of 4.1 out of 5. Cognitive participation (NPT Component 2) was examined by question 7. The mean score for cognitive participation was 3.1 out of 5. Collective action (NPT Component 3) was evaluated through questions 8–10. Participants scored the intervention an average of 3.2 out of 5 for collective action. Reflexive monitoring (NPT Component 4) was assessed through questions 11–13. Participants gave iSupport RDC an average score of 3.5 out of 5 for this component.

#### Content analysis of qualitative feedback

Nineteen out of the 24 participants provided qualitative feedback in response to questions “Did you have problems accessing the iSupport website?—If yes please describe –” and “Do you have any additional feedback on iSupport for Rare Dementias?” as a part of the post-intervention questionnaire. A content analysis table was created (Additional file 4), six codes were identified, and their frequencies calculated (Table 4). The most frequently reported category was technical difficulties which were reported by ten participants, seven participants made comments about how iSupport RDC is useful and informative, five specified that it should be provided at the point of diagnosis to reach the people that need the information the most, three participants gave additional information to add to iSupport RDC, three also said they would have preferred a different format (paper), and one participant said that it was difficult to integrate into life as a carer. To address this feedback, iSupport RDC is now hosted on a different platform, with improved accessibility. The information that was suggested as important to add (Table 4) has been reviewed by the research team and added, and iSupport RDC is available as a PDF so participants can print it out as a paper copy if that is their preference.

**Table 4:** Categories of qualitative feedback identified using content analysis and the frequency of each

Category	Frequency	Description	Example quote
Provide iSupport at the point of diagnosis	5	iSupport RDC would be useful for people sooner after diagnosis. Recruiting through the RDS meant that participants already have access to a lot of information and several participants said that they had been caring for a long time so learnt these tips from other sources	“iSupport wasn’t particularly relevant for me; the reason for this is that my husband is now in his sixth year since diagnosis and I already have found out or researched the (albeit very useful) information long ago, so I am already familiar with all the strategies and suggestions the tool provides. Having said that, I know I would have been very grateful for something like this when we were at the start of this journey, when I found it virtually impossible to get the help and information we needed.”
Useful and informative	7	iSupport RDC gave them useful information, that the external links were useful, it was not patronising and very relevant for carers of people with rare dementias	“I found it easy to follow. It was also really refreshing, it wasn’t condescending. It felt supportive & creative & enabled space in challenging situations.”
Technical difficulties	10	Problems logging in, navigating the platform or other IT related problems were reported. These problems include incompatibility with browsers or operating systems and difficulties following the login process	“I have tried a couple of times to log on and it says my browser doesn’t allow TDM to function so needed the PDF.”
Information to add or expand on	3	Suggestions to add additional information to iSupport RDC were made, including information on Charles Bonnet syndrome, more about planning ahead, reassurance that carers can cope and emphasis on the fact that although rare dementias are more likely to occur in people below the age of 65, they do occur in older adults as well	“I would have liked a bit more about planning for the future and what might come next, knowing we can cope and being prepared and confident etc.”
Different format would be preferred	3	Preference for a different format was expressed: a paper copy, the PDF version, and online support was not seen as useful	“My only “criticism”? I would like this in printed and bound form, as a companion manual to sit alongside me and my coffee; I’d buy one.”
Difficult to integrate into life as a carer	1	Difficulties with integrating iSupport RDC into life as a carer and that reminders might be helpful	“I find it difficult to integrate it into my life as a carer. Along with everything else I needed to do. Maybe a nudge would work for me. I try to make time for everything, but something always slips.”

### Secondary outcome measures

The data for all four measures were not normally distributed and the standard deviations were large, so median scores were used instead of means (Table 5) for the 24 participants who completed both pre- and post-intervention measures.

**Table 5:** Median (Mdn) and interquartile ranges (IQR) of pre- and post-intervention scores for anxiety, depression, burden, and resilience (*N* = 24)

	Pre		Post		CI (95%)	
	Mdn	IQR	Mdn	IQR	Lower	Upper
Anxiety (GAD-7)	6	6.8	4	5.3	− 0.16	0.62
Depression (CES-D-10)	12	7.5	11	5.5	− 0.22	0.56
Burden (ZBI-12)	22	8.5	23	9.5	− 0.40	0.39
Resilience (RS-14)	75	19	79	18.5	− 0.66	0.14

Confidence intervals (CIs) represent the CI for the difference in medians

Calculated using the standard error of the median and the normal approximation method

**Fig. 2:**
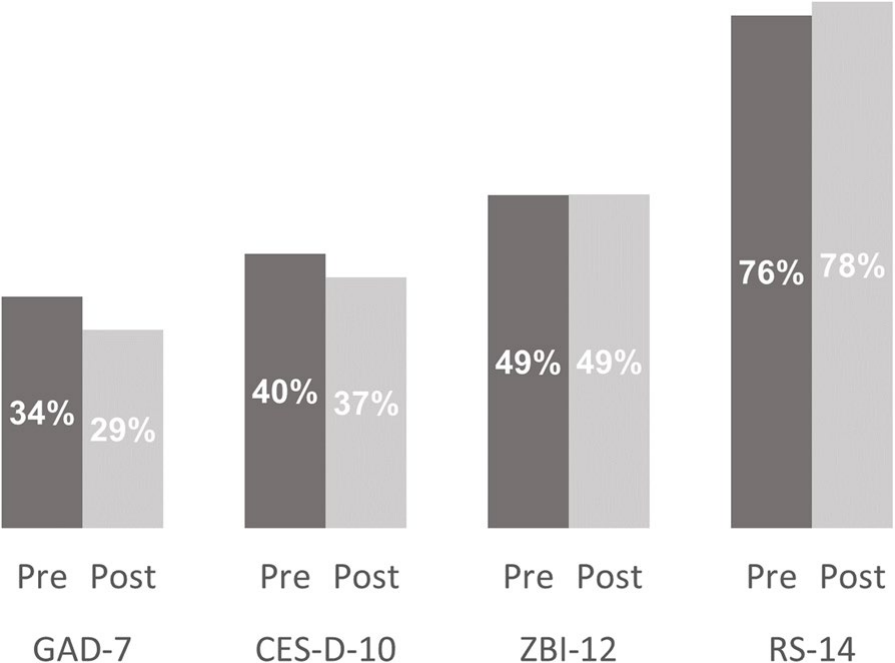
Median percentage scores pre- and post-intervention

The median percentage scores were calculated to summarize the pre- and post-intervention values for each measure (Fig. 2). Anxiety and depression scores showed slight decreases, resilience showed a slight increase, and burden remained unchanged. However, given the small sample size and the feasibility nature of this study, these variations should be interpreted with caution, as they are subject to small sample fluctuations. The 95% confidence intervals all include zero, indicating that these results are consistent with no change. Further research with a larger sample and longer intervention period is needed to explore the potential impact of iSupport RDC on carer wellbeing.

## Discussion

This study examined the feasibility of the online iSupport RDC programme, focussing on the feasibility of recruitment, the feasibility of the measurement tools, and the acceptability of iSupport RDC ahead of any future large-scale study. The findings were promising but also highlight several limitations that should be considered in any further studies.

Participant recruitment exceeded the target number of 30 participants, however half of those invited to take part in the study declined. The attrition rate was moderate, with 24 out of the 34 recruited participants completing the study over the three months resulting in a completion rate of 70.6%.

The withdrawal of ten participants from the study revealed noteworthy trends: eight of these individuals were female, eight belonged to the age bracket of 60–79 years, and seven had attained education at a degree level or higher. This gender disproportion in withdrawals contradicts established research suggesting that women tend to seek advice online more frequently [37]. Additionally, the Indian iSupport adaptation study discovered that female participants engaged with iSupport India modules more extensively than their male counterparts [38]. While this unexpected gender skew in withdrawals could potentially be attributed to individual differences in digital literacy or unique personal circumstances, it might also be linked to the initially higher representation of female carers recruited for this study. However, future research could explore gender-specific needs within caregiving roles. Furthermore, the ages of the carers who withdrew align with prior findings indicating that the older carers are less likely to engage with eHealth technology [39].

The higher-than-expected dropout rate among highly educated participants, seven of the ten withdrawals, warrants further investigation. While higher education is typically associated with greater technology use [37], this finding suggests that other factors may influence engagement. Highly educated carers may have different expectations of digital platforms, or they may face particular challenges that influence their participation. These could include a more critical evaluation of the platform’s relevance to their needs. This unexpected pattern emphasises the need to explore how factors such as educational background, caregiving expectations, and platform usability interact to affect participant retention [39].

Higher resilience among withdrawn participants in this study suggests that these individuals may possess unique coping mechanisms that allow them to manage caregiving challenges despite feelings of isolation or withdrawal. This contrasts with the trends observed in anxiety, depression, and burden, which generally reflect higher levels of emotional distress and caregiving strain. The discrepancy between high resilience and elevated anxiety, depression, and burden may indicate that resilience acts as a buffer or protective factor in coping with caregiving demands, potentially allowing withdrawn participants to endure stressors while appearing emotionally disengaged. These findings highlight the complex nature of caregiving, where resilience does not necessarily correlate with the absence of psychological distress.

The completion rate for this study (70.6%) was higher than the completion rate in the iSupport study in India (36.42%) [38], but not as high as the Portuguese adaptation (73.8%) [40]. The sample size in this study was smaller than both samples for the Portuguese adaptation [41] and the Indian adaptation (151), so the completion rate may change with more participants. To retain participants with higher psychological distress, it may be beneficial to offer additional support, such as referrals to the RDS support services to address their emotional needs. The offer of technical support from the researcher was accepted by some participants but the participants that withdrew did not respond to these offers. An online social support forum, as suggested in other iSupport studies [42] may also provide an accessible platform for participants to connect with others facing similar challenges, fostering a sense of community and reducing feelings of isolation, ensuring participants feel valued and motivated to remain involved in the study.

There was a lack of diversity in the participants, as thirty-three were White and 59% had received a degree level qualification or higher. Any future study would need to make efforts to recruit a more diverse sample of participants. The lack of diversity may be due to the British RDS charity participant pool being predominately White, however, it is well documented that dementia research in high-income countries has a lack of ethnic diversity [41]. Prevalence rates of rare dementias differ among ethnic groups. LBD has higher prevalence rates in white people than Black people, Asian and Pacific Islanders and Latino people [43]. FTD is less common in Black and Latino people than White people whereas the rates are comparable to Asian and Pacific Islanders [43]. Although LBD and FTD are more commonly diagnosed in White people, their carers may not necessarily be from the same ethnic group. So, testing iSupport RDC with an ethnically diverse sample is necessary. Previous research has found that White British carers described a need for more educational resources for new carers, which British South Asian carers did not mention when asked about the support that they would like to receive [44]. If White carers prefer educational resources, then this could be an alternative reason behind the lack of cultural diversity in the study participants. The lack of ethnic diversity in this study limits the generalisability of the findings. Future studies should prioritise inclusive recruitment strategies, such as partnering with community organisations, using culturally tailored outreach materials, and leveraging social media to reach a broader audience. Ensuring that recruitment efforts specifically target carers from diverse ethnic, socioeconomic, and geographical backgrounds will provide more representative data and enhance the relevance of the intervention across varied caregiving contexts.

Fifty-nine percent of the participants had a degree level qualification or higher, this is higher than the UK population where 33.8% of the population is educated to a degree level or higher [45]. This suggests that the RDS sample is a highly educated population and future research should aim to test iSupport RDC on a sample with varying levels of education.

To assess the feasibility of measurement tools, missing responses and time taken to finish the questionnaires were assessed. The median time taken to complete the questionnaires was around 8 min, and coupled with the lack of missing responses suggested the measurement tools would be feasible to use in a larger scale study. All measures were completed fully with no missing answers. except for one participant, who recently had a bereavement and who found the questionnaires more difficult to complete. Future studies should consider this potential circumstance and ensure protocols are put in place to support carers, to either support them to withdraw or continue with the study as they feel appropriate, but also ensure all items on the measurements are still relevant. This should involve checking the tense of the questions and whether any of them refer to the person with dementia having passed away. Another unexpected outcome emerged when a participant, upon completing wellbeing measures, experienced a self-reflective process. This led to the unintended identification of their mental health challenges, prompting voluntary withdrawal due to a perceived unsuitability to participate. This unanticipated outcome highlights the importance of considering psychological impacts and unintended consequences of assessment tools, even in pilot trials, emphasising our ethical responsibility to mitigate harm and prioritise participant well-being.

The findings from the modified NoMAD questionnaire [21, 23] suggest that the participants in this study were able to make sense of the intervention, demonstrated commitment, engaged in collective actions, and engaged in reflexive monitoring. Participants gave iSupport RDC a mean score of 3.5/5 across the four components of NPT suggesting that it showed promise in terms of participants’ engagement [22]. Guidelines on using the NoMAD state that scores should be interpreted as positive or negative, with any score above 2.5 seen as a positive result. The component with the lowest score was cognitive participation (3.1), this refers to the commitment and engagement of the participants. Based on the qualitative feedback, many of the participants felt that it was not relevant for them at their stage of caring, which could explain this score. However, a score of 3.1 out of 5 is above 2.5 and therefore indicates a positive outcome.

Qualitative feedback from the participants was mainly positive. One participant mentioned that a reminder to complete the modules would have been useful. Since the intervention is made to be flexible and used whenever needed, the reminder may be useful in the context of a study when there is a time limit rather than in a real-world clinical context. It was mentioned by a few participants that iSupport RDC would be useful for carers sooner after diagnosis, so the early availability of the intervention is something to consider in the future. This earlier access was also mentioned by carers in the adaptation phase of the project [14]. Participants suggested several minor edits, which were subsequently implemented, as guidelines on intervention development describe the process as an ongoing cycle of adapting, testing, and improving interventions [46, 47]. These edits including information about the Charles Bonnet syndrome, more advice on planning ahead, and acknowledgement that although rare dementias are more likely to occur in people below the age of 65, they do occur in older adults as well.

The main limit in the acceptability of iSupport was the presence of IT-related problems reported by ten participants. Technical issues such as slow internet connections, difficulties navigating the platform, and incompatibility with internet browsers and operating systems raised concerns about the feasibility and accessibility of the online iSupport intervention. These issues impacted participants’ engagement and adherence to the program and caused four people to withdraw from the study, which could have influenced the magnitude of the observed effects. Blackboard Learn was meant to provide usability data including how frequently participants logged on, the duration spent logged on and the duration spent on each module. Unfortunately, this data were unreliable and gave conflicting results, consequently, it was not used in the analysis and a correlation between time spent on iSupport RDC and the improvements in secondary outcome measures was not calculated as planned. An alternative host website that helps mitigate these issues should be used for any further testing and implementation of the iSupport intervention. iSupport RDC is now available on a new host platform, eliminating the need for a login process, which simplifies access for users. Accessibility has been further enhanced with read-aloud functions for individuals with visual impairments, and the platform is also available as a downloadable PDF, ensuring flexibility for users with limited internet access. Offering multilingual options could further expand the platform’s reach, effectively supporting diverse caregiving communities.

### Strengths and limitations

Despite the positive feedback from participants, several limitations of the study must be acknowledged. A limitation of this study was the lack of diversity among the participants. Rare dementias affect people worldwide, and variations in coping mechanisms, social support needs, and perceptions of online interventions may play a significant role in the acceptability of the intervention. Studies on dementia care often end up with a majority, if not all female participants [10]. The participants for this study were 38% male which is positive considering an estimated 19% of informal carers globally are male [1].

One limitation of our study is the absence of established progression criteria. While this approach allowed for a more comprehensive and flexible evaluation of feasibility factors, without predefined benchmarks, it can be challenging to objectively determine the success or failure of various study components.

### Future directions

Future research should focus on addressing the previously mentioned limitations to further explore the potential of the online iSupport RDC intervention. Strategies to address and avoid IT problems, could include more user-friendly platforms that do not require log-in processes or detailed instructions to use. Rigorous testing on multiple web browsers and operating systems is also required. Efforts to include participants from diverse cultural backgrounds are essential to assess the intervention’s cross-cultural applicability and effectiveness. Studies with larger sample sizes are warranted to enhance the robustness of findings and identify the effects of iSupport on burden, depression, anxiety and resilience. Finally, to better meet participants’ needs, iSupport RDC could be tailored to address the unique challenges faced at different caregiving stages. Early-stage carers may benefit from modules focused on understanding rare dementias and navigating initial diagnoses, while later-stage carers might require more targeted guidance on managing advanced symptoms and self-care strategies. Feedback from carers in the adaptation study [14] suggested that incorporating a search bar could make it easier for users with diverse needs to locate relevant content, further enhancing the platform’s accessibility and usability. Personalised content pathways or adaptable modules could ensure iSupport RDC remains responsive to the evolving needs of carers throughout their journey.

## Conclusions

This study provides initial evidence of the feasibility of the online iSupport RDC intervention, with positive trends on resilience, depression, and anxiety, along with qualitative feedback describing its usefulness. The measurement tools also demonstrated feasibility for a larger scale study. The results align with broader trends in digital health, where eHealth interventions offer accessible and scalable support for carers. However, it is important to acknowledge the limitations associated with IT problems and a lack of cultural diversity in participants. These limitations should guide future research into the iSupport intervention or other online interventions to better support carers facing the challenges of rare dementias. Freely accessible at www.isupportdementiacarers.co.uk, iSupport RDC has the potential to address the growing demand for caregiver resources by providing tailored, evidence-based support remotely.

## Supplementary Information

Additional file 1. CONSORT extension to pilot and feasibility trials checklist.

Additional file 2. Login instructions provided to participants.

Additional file 3. Modified NoMAD questionnaire in English and Welsh

Additional file 4. Content Analysis table.

## Data Availability

The participants of this study did not give written consent for their data to be shared publicly, so due to the sensitive nature of the research supporting data is not available.

## Abbreviations

PwD: Person with dementia
WHO: World Health Organisation
RDS: Rare Dementia Support
iSupport RDC: iSupport for rare dementia carers
FTD: Frontotemporal dementia
PCA: Posterior cortical atrophy
PPA: Primary progressive aphasia
LBD: Lewy body dementia
NPT: Normalisation Process Theory
GAD- 7: Generalised anxiety disorder questionnaire
CES-D- 10: Center for Epidemiologic Studies Depression Scale
ZBI- 12: Zarit Burden Inventory
RS- 14: Resilience Scale
