# Supplementary material for: iSupport for rare dementias: a mixed-methods non-randomised feasibility study of an online self-help programme for carers
Source: Pilot Feasibility Stud. 2025 Apr 30;11:58. doi: 10.1186/s40814-025-01639-z (PMC12042611; doi:10.1186/s40814-025-01639-z)

Accessing iSupport for Rare Dementias

You will receive an email containing a username (email address) and password. This is unique to you and nobody else can access your iSupport account.

Step 1-Click on the following link: [Prifysgol Bangor University]()

Step 2 -Click “sign in” in the top right corner

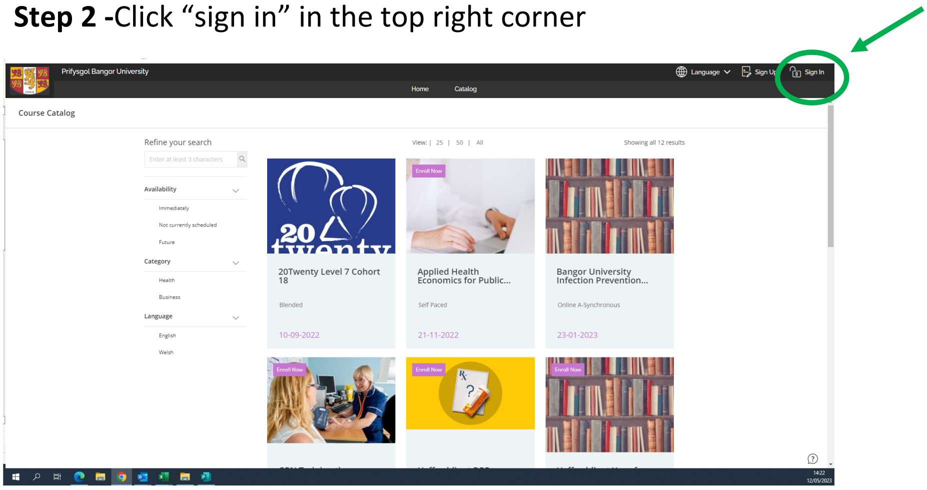

Step 3- Enter the username and password that have been sent to you

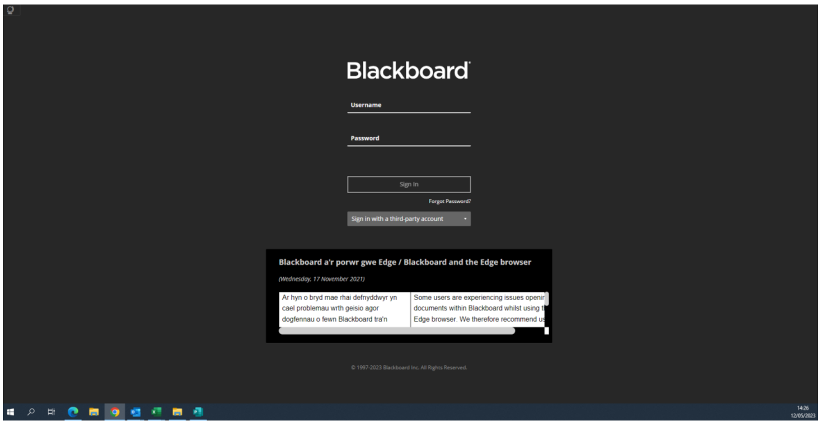

Step 4– Click on My Dashboard at the top

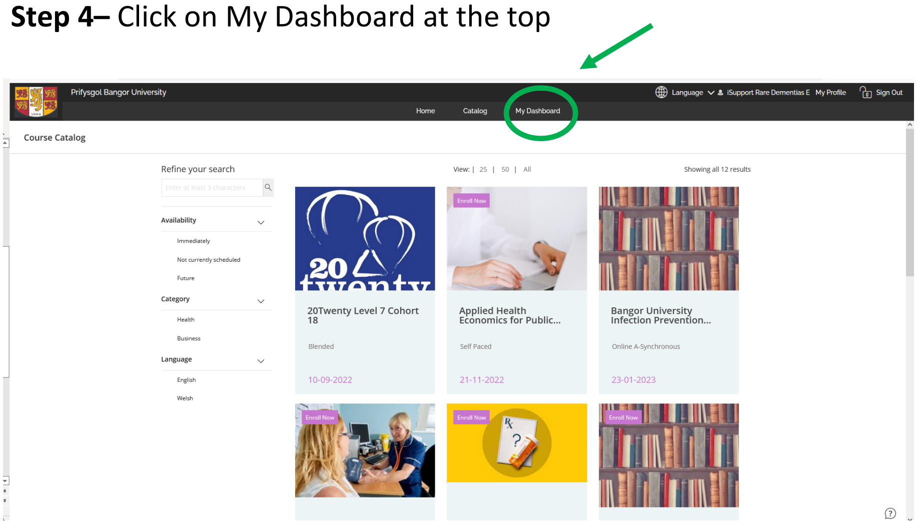

Step 5– Click on Continue Course

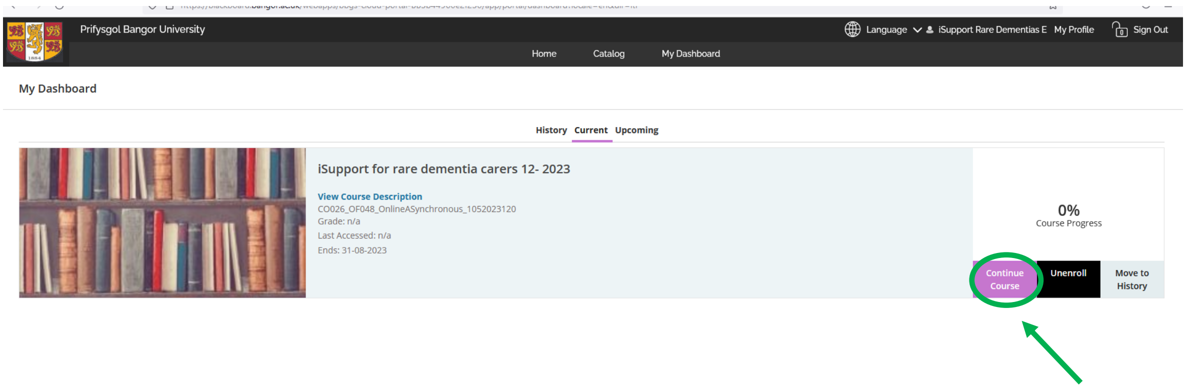

Step 6– Select the module you want look at and use the arrows to navigate forward

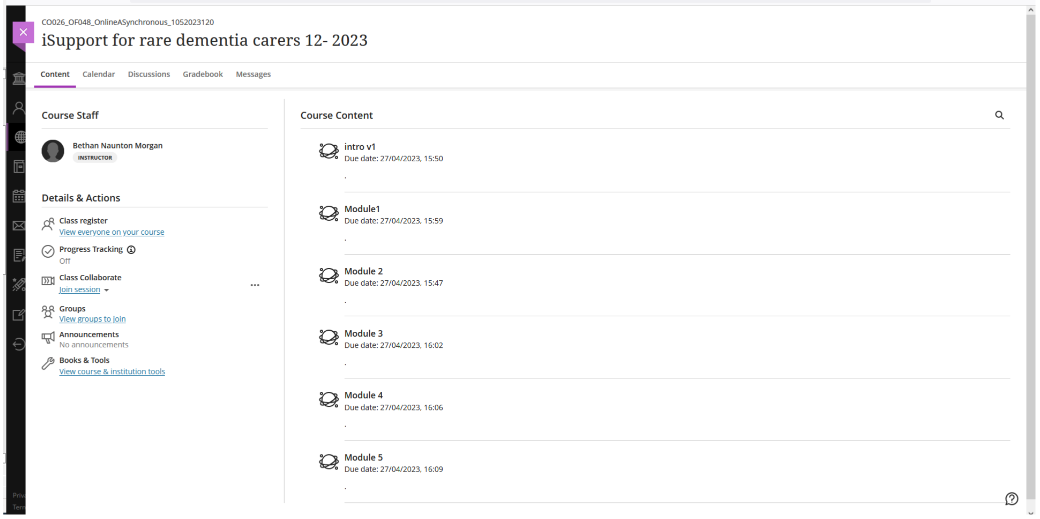

Supplement: Supplementary file 2 — Additional file 2. Login instructions provided to participants. [file 40814_2025_1639_MOESM2_ESM.pdf]
