# Supplementary material for: iSupport for rare dementias: a mixed-methods non-randomised feasibility study of an online self-help programme for carers
Source: Pilot Feasibility Stud. 2025 Apr 30;11:58. doi: 10.1186/s40814-025-01639-z (PMC12042611; doi:10.1186/s40814-025-01639-z)
Supplement: Supplementary file 3 — Additional file 3. Modified NoMAD questionnaire in English and Welsh [file 40814_2025_1639_MOESM3_ESM.docx]

NPT Eng

Please take the time to decide which answer best suits your experience for each statement and tick the appropriate circle.

1. When you use iSupport for Rare Dementias, how familiar does it feel?

- Not familiar at all
- Slightly familiar
- Moderately familiar
- Very familiar
- Extremely familiar

2. Do you feel iSupport for Rare Dementias is currently a normal part of your routine?

- Definitely not
- Probably not
- Might or might not be
- Probably yes
- Definitely yes

3. Do you feel iSupport for Rare Dementias will become a normal part of your routine?

- Definitely not
- Probably not
- Might or might not be
- Probably yes
- Definitely yes

4. I can see how iSupport for Rare Dementias differs from other support packages

- Strongly disagree
- Somewhat agree
- Neither agree nor disagree
- Somewhat agree
- Strongly agree

5. I understand the purpose of iSupport for Rare Dementias

- Strongly disagree
- Somewhat agree
- Neither agree nor disagree
- Somewhat agree
- Strongly agree

6. I can see the potential value of iSupport of Rare Dementias for carers of people with a rare dementia

- Strongly disagree
- Somewhat agree
- Neither agree nor disagree
- Somewhat agree
- Strongly agree

7. I will continue to use iSupport for Rare Dementias

- Strongly disagree
- Somewhat agree
- Neither agree nor disagree
- Somewhat agree
- Strongly agree

8. I can easily integrate iSupport for Rare Dementias into my day

- Strongly disagree
- Somewhat agree
- Neither agree nor disagree
- Somewhat agree
- Strongly agree

9. I have confidence in other people’s ability to use iSupport for Rare Dementias

- Strongly disagree
- Somewhat agree
- Neither agree nor disagree
- Somewhat agree
- Strongly agree

10. Sufficient resources (staff) are available to support iSupport for Rare Dementias

- Strongly disagree
- Somewhat agree
- Neither agree nor disagree
- Somewhat agree
- Strongly agree

11. I value the effects that iSupport for Rare Dementias has had on me

- Strongly disagree
- Somewhat agree
- Neither agree nor disagree
- Somewhat agree
- Strongly agree

12. I have changed how I deal with situations based on iSupport for Rare Dementias

- Strongly disagree
- Somewhat agree
- Neither agree nor disagree
- Somewhat agree
- Strongly agree

13. Feedback about iSupport for Rare Dementias can be used to improve it in the future

- Strongly disagree
- Somewhat agree
- Neither agree nor disagree
- Somewhat agree
- Strongly agree

NPT Cym

Cymerwch eich amser i benderfynu pa ateb sy'n disgrifio eich profiad orau yn achos pob gosodiad a thiciwch y cylch priodol.

1. Pan fyddwch yn defnyddio iSupport ar gyfer Mathau Prin o Ddementia, pa mor gyfarwydd y bydd yn teimlo?

- Ddim yn gyfarwydd o gwbl
- Ychydig yn gyfarwydd
- Gweddol gyfarwydd
- Cyfarwydd iawn
- Hynod o gyfarwydd

2. Ydych chi'n teimlo bod iSupport ar gyfer Mathau Prin o Ddementia yn rhan o'ch trefn arferol ar hyn o bryd?

- Yn bendant ddim
- Mwy na thebyg ddim
- Efallai neu efallai ddim
- Ydy, mwy na thebyg
- Ydy, yn bendant.

3. Ydych chi'n teimlo y bydd iSupport ar gyfer Mathau Prin o Ddementia yn tyfu’n rhan o'ch trefn arferol?

- Yn bendant ddim
- Mwy na thebyg ddim
- Efallai neu efallai ddim
- Bydd, mwy na thebyg
- Bydd, yn bendant.

4. Gallaf weld sut mae iSupport ar gyfer Mathau Prin o Ddementia yn wahanol i becynnau cefnogi eraill

- Anghytuno’n gryf
- Cytuno i raddau
- Ddim yn cytuno nac yn anghytuno
- Cytuno i raddau
- Cytuno’n gryf

5. Rwy'n deall pwrpas iSupport ar gyfer Mathau Prin o Ddementia

- Anghytuno’n gryf
- Cytuno i raddau
- Ddim yn cytuno nac yn anghytuno
- Cytuno i raddau
- Cytuno’n gryf

6. Gallaf weld gwerth posibl iSupport ar gyfer Mathau Prin o Ddementia i ofalwyr pobl sydd â math prin o ddementia

- Anghytuno’n gryf
- Cytuno i raddau
- Ddim yn cytuno nac yn anghytuno
- Cytuno i raddau
- Cytuno’n gryf

7. Byddaf yn parhau i ddefnyddio iSupport ar gyfer Mathau Prin o Ddementia

- Anghytuno’n gryf
- Cytuno i raddau
- Ddim yn cytuno nac yn anghytuno
- Cytuno i raddau
- Cytuno’n gryf

8. Gallaf integreiddio iSupport ar gyfer Mathau Prin o Ddementia yn hawdd i’m diwrnod

- Anghytuno’n gryf
- Cytuno i raddau
- Ddim yn cytuno nac yn anghytuno
- Cytuno i raddau
- Cytuno’n gryf

9. Mae gen i hyder yng ngallu pobl eraill i ddefnyddio iSupport ar gyfer Mathau Prin o Ddementia

- Anghytuno’n gryf
- Cytuno i raddau
- Ddim yn cytuno nac yn anghytuno
- Cytuno i raddau
- Cytuno’n gryf

10. Mae adnoddau digonol (staff) ar gael i gefnogi iSupport ar gyfer Mathau Prin o Ddementia

- Anghytuno’n gryf
- Cytuno i raddau
- Ddim yn cytuno nac yn anghytuno
- Cytuno i raddau
- Cytuno’n gryf

11. Rwy'n gwerthfawrogi'r effeithiau y mae iSupport ar gyfer Mathau Prin o Ddementia wedi'u cael arnaf

- Anghytuno’n gryf
- Cytuno i raddau
- Ddim yn cytuno nac yn anghytuno
- Cytuno i raddau
- Cytuno’n gryf

12. Rwyf wedi newid sut y byddaf yn ymdrin â sefyllfaoedd yn sgil iSupport ar gyfer Mathau Prin o Ddementia

- Anghytuno’n gryf
- Cytuno i raddau
- Ddim yn cytuno nac yn anghytuno
- Cytuno i raddau
- Cytuno’n gryf

13. Gellid defnyddio adborth ynglŷn ag iSupport ar gyfer Mathau Prin o Ddementia i'w wella yn y dyfodol

- Anghytuno’n gryf
- Cytuno i raddau
- Ddim yn cytuno nac yn anghytuno
- Cytuno i raddau
- Cytuno’n gryf
